# Supplementary material for: Tumor Necrosis Factor-Alpha Inhibitor Use and Malignancy Risk: A Systematic Review and Patient Level Meta-Analysis
Source: Cancers (Basel). 2025 Jan 24;17(3):390. doi: 10.3390/cancers17030390 (PMC11815771; doi:10.3390/cancers17030390)
Supplement: Supplementary file 1 [file cancers-17-00390-s001.zip › cancers-3364476-supplementary.pdf]

Supplementary Material

Methods S1: PubMed MEDLINE Search Strategy

Search strategy in PubMed MEDLINE (example for Infliximab):

(Infliximab OR Avakine OR Flixabi OR Inflectra OR Ixifi OR Remicade OR Remsima OR Renflexis OR Revellex OR Zessly)

AND

((safe OR safety OR "side effect" OR "side effects" OR "undesirable effect" OR "undesirable effects" OR "treatment emergent" OR tolerability OR toxicity OR adrs OR Neoplasms[Mesh] OR neoplas* OR cancer OR cancers OR cancerous OR carcinogen* OR malignan* OR oncolog* OR ((adverse OR long-term OR "long term") AND (effect OR effects OR reaction OR reactions OR event OR events OR outcome OR outcomes)))))

AND

(longitudinal studies [MeSH] OR “time factors” [MeSH] OR cumulative OR duration* OR long-term OR short-term OR “long term” OR “short term” OR "longer term" OR "shorter term" OR longitudinal OR continuous OR continual OR aggregate OR follow-up OR "follow up" OR timepoints OR time-points OR accumulate* OR "treatment period" OR "latency period" OR "latency periods" or "study period" OR interval OR intervals)

**MeSH Term**	**MeSH Unique ID**
Infliximab [MeSH]	D000069285
Adalimumab [MeSH]	D000068879
Etanercept [MeSH]	D000068800
Certolizumab Pegol [MeSH]	D000068582
Golimumab [Supplementary Concept]	C529000
Neoplasms [MeSH]	D009369
Longitudinal Studies [MeSH]	D008137
Time Factors [MeSH]	D013997

Supplementary Table S1: Malignancy Incidence of Interventional and Observational Studies of Patients Exposed to TNF-I

	Author, Year	Total # TNF Cancers	Total # of TNF Cancers Excluding NMSC	Cancer Info	Total # Placebo Cancers	Total # Placebo Cancers Excluding NMSC	Cancer Information
	INTERVENTIONAL						
ADA							
	Bissonnette, 2017 [47]	1	1	1 Bladder	0	0	None
	Cai, 2016 [48]	2	2	1 Uterine, 1 Gastric	0	0	None
	Colombel, 2014 [49]	25	25	22 Unspecified, 3 Lymphoma	2	2	2 Unspecified
	Furst, 2003 [50]	1	1	1 NHL	0	0	None
	Genovese, 2007 [51]	0	0	None	3	2	1 NHL, 1 Prostate, 1 NMSC
	Jaffe, 2016 [52]	2	2	1 GI Carcinoid, 1 GBM	0	0	None
	Kamm, 2011 [53]	6	6	6 Unspecified	0	0	None
	Kimball, 2016 [54]	1	0	1 NMSC[%]	1	1	1 Breast
	Leonardi, 2011 [55]	0	0	None	1	1	1 Breast
	Menter, 2010 [56]	6	2	4 NMSC, 2 Unspecified	2	1	1 NMSC, 1 Unspecified
	Miyasaka, 2008 [57]	0	0	None	2	2	2 Unspecified
	Mostafa, 2017 [58]	8	2	6 NMSC, 2 Unspecified	0	0	None
	Reinisch, 2011 [59]	0	0	None	2	1	1 Breast, 1 NMSC
	Suzuki, 2017 [60]	8	8	1 Pancreatic, 7 Unspecified	0	0	None

	van de Putte, 2004 [61]	4	4	1 Cholangiocarcinoma, 1 Adenocarcinoma, 2 Unspecified	1	1	1 Unspecified
	van der Heijde, 2009 [62]	4	2	1 Melanoma, 1 NHL, 2 NMSC	0	0	None
	Gordon, 2006 [63]	5	5	1 Breast, 1 Gastric, 2 Melanoma, 1 Oral SCC	0	0	None
INX							
	Kavanaugh, 2007 [64]	1	1	1 HL	1	0	1 NMSC
	Koninckx, 2008 [65]	1	1	1 Leukemia	0	0	None
	Mariette, 2004 [66]	1	1	1 Breast	0	0	None
	Menter, 2007 [67]	12	2	1 Breast, 1 Ovarian, 10 NMSC	0	0	None
	Rennard, 2007 [68]	12	12	1 Kidney, 1 Breast, 6 Lung, 1 Pancreatic, 1 HL, 2 Laryngeal SCC	3	3	2 Prostate, 1 Cervical
	Rutgeerts, 1999 [69]	1	1	1 NHL	0	0	None
	Reinisch, 2012 [70]	5	3	1 Prostate, 1 Lung, 1 Breast, 2 NMSC	0	0	None
	Rutgeerts, 2005 [71]	2	1	1 Prostate, 1 NMSC	0	0	None
	Rutgeerts, 2005 [71]	1	1	1 Colorectal	1	0	1 NMSC
	Regueiro, 2016 [72]	0	0	None	2	2	2 Unspecified
	Sandborn, 2004 [73]	1	1	1 Cervical	1	1	1 Colorectal
	Braun, 2008 [74]	4	3	1 Testicular, 1 Breast, 1 Lung, 1 NMSC	0	0	None
ETN							
	Dijkmans, 2009 [75]	4	3	1 Melanoma, 1 NMSC, 1	0	0	None

				Unspecified, 1 Pituitary tumor			
	Kloppenburg, 2018 [76]	1	1	1 Breast	0	0	None
	Tyring, 2007 [77]	23	9	2 Breast, 1 Tonsil SCC, 1 Lung, 2 Colorectal, 1 Pancreatic, 1 HL, 1 NHL, 14 NMSC	0	0	None
	Weisman, 2007 [78]	2	0	2 NMSC	3	2	1 Lung, 1 NMSC, 1 Unspecified
GLM							
	Bao, 2014 [79]	1	1	1 Ovarian Cancer	0	0	None
	Deodhar, 2015 [80]	4	2	1 Pancreatic cancer, 1 Lymphoma, 2 NMSC	0	0	None
	Husni, 2020 [81]	2	2	1 Colorectal, 1 Gastric	2	2	1 Lung, 1 Esophageal
	Takeuchi, 2017 [82]	5	5	2 Ovarian, 1 Colorectal, 1 Breast, 1 Pancreatic	0	0	None
	Wenzel, 2009 [83]	8	6	1 Kidney, 1 Breast, 1 Colorectal, 1 Cervical, 1 Melanoma, 1 NHL, 2 NMSC	0	0	None
	Emery, 2013 [84]	1	1	1 Breast	3	1	1 Breast, 2 NMSC
	Kremer, 2010 [85]	2	1	1 Colorectal, 1 NMSC	2	1	1 Thyroid, 1 NMSC
CTZ							
	Mease, 2014 [86]	1	1	1 Cervical	0	0	None
	Sandborn, 2007 [87]	2	2	1 Lung, 1 Colorectal	2	2	1 Cervical, 1 HL

	Weinblatt, 2015 [88]	2	2	2 Lung	0	0	None
	Sandborn, 2011 [89]	1	1	1 Metastatic adenocarcinoma	0	0	None
	Loftus Jr, 2016 [90]	35	22	1 NHL, 4 Melanoma, 13 NMSC, 17 Unspecified	3	3	1HL, 2 Unspecified
	Yamamoto, 2014 [91]	0	0	None	1	1	1 Unspecified
	OBSERVATIONAL						
ADA							
	Klotsche, 2016 [92]	2	2	1 NHL, 1 Oligodendroglioma	2	2	2 ALL
	Mercer, 2017 [25]	34	34	4 HL, 30 NHL	30	30	5 HL, 25 NHL
	Lunder, 2019 [93]	8	8	8 Unspecified	3	3	3 Unspecified
	Viguier, 2012 [94]	1	1	1 NHL	0	0	None
	Mason, 2018 [95]	11	11	11 Unspecified	13	13	13 Unspecified
INX							
	Lunder, 2019 [93]	1	1	1 Unspecified	3	3	3 Unspecified*
	Mok, 2014 [96]	4	4	4 Unspecified	0	0	None
	Nossent, 2001 [97]	1	1	1 Prostate	0	0	None
	Sakai, 2012 [98]	6	6	6 Unspecified	1	1	1 Unspecified
	Rennard, 2012 [99]	20	20	2 Prostate, 1 Kidney, 1 Breast, 10 Lung, 1 Colorectal, 1 Lymphoma, 3 Head and Neck, 1 Unspecified	8	8	2 Lung, 5 Prostate, 1 Unspecified
ETN							
	Driessen, 2009 [100]	7	2	1 Breast, 1 Esophageal, 5 NMSC	1	0	1 NMSC

	Lunder, 2019 [93]	2	2	2 Unspecified	3	3	3 Unspecified*
	Mason, 2018 [95]	7	7	7 Unspecified	13	13	13 Unspecified*
	Mok, 2014 [96]	6	6	6 Unspecified	0	0	None
	Sakai, 2012 [98]	3	3	3 Unspecified	1	1	1 Unspecified*

*appears earlier in table
^Adalimumab (ADA), Infliximab (INX), Etanercept (ETN), Certolizumab (CTZ), Golimumab (GLM)
%Non-melanoma skin cancer (NMSC, basal or squamous cell carcinoma of the skin excluded due to lack of metastatic potential

Supplementary Table S2: Risk of Bias Assessments: A) Cochrane (Randomized); B) ROBINS-I (Non-Randomized)

A)

		Random sequence generation: will randomization produce comparable groups?	Allocation concealment: determine whether intervention allocations could've been foreseen during or before enrollment.	Blinding of participants and personnel	Attrition bias: incomplete outcome data	Reporting bias: selective outcome reporting	Other bias
ADA							
	Bissonnette, 2017 [47]	Low risk (high-quality randomization)	Low risk	Low risk	Unclear risk	Low risk	Unclear risk
	Cai, 2016 [48]	Unclear risk	Low risk	Low risk	Unclear risk	Low risk	Unclear risk
	Colombel, 2014 [49]	Low risk (high-quality randomization)	Low risk	Low risk	Low risk	Unclear risk	Unclear risk

	Furst, 2003 [50]	Low risk (high-quality randomization)	Unclear risk	Low risk	Unclear risk	Unclear risk	Low risk
	Genovese, 2007 [51]	Low risk (high-quality randomization)	Unclear risk	Low risk	Unclear risk	Low risk	Unclear risk
	Jaffe, 2016 [52]	Low risk (high-quality randomization)	Low risk	Low risk	Unclear risk	Unclear risk	Unclear risk
	Kamm, 2011 [53]	Low risk (high-quality randomization)	Low risk	Low risk	Unclear risk	Unclear risk	Low risk
	Kimball, 2016 [54]	Low risk (high-quality randomization)	Unclear risk	Low risk	Unclear risk	Unclear risk	Unclear risk
	Leonardi, 2011 [55]	Low risk (high-quality randomization)	Unclear risk	High risk	Unclear risk	Low risk	Unclear risk
	Menter, 2010 [56]	Low risk (high-quality randomization)	Unclear risk	Low risk	Unclear risk	Low risk	Unclear risk
	Miyasaka, 2008 [57]	Low risk (high-quality randomization)	Unclear risk	Low risk	Unclear risk	Unclear risk	Low risk
	Mostafa, 2017 [58]	Low risk (high-quality randomization)	Unclear risk	Unclear risk	Low risk	Unclear risk	Unclear risk
	Reinisch, 2011 [59]	Low risk (high-quality randomization)	Unclear risk	Low risk	Unclear risk	Low risk	Unclear risk
	Suzuki, 2017 [60]	Low risk (high-quality randomization)	Unclear risk	Low risk	Unclear risk	Low risk	Unclear risk
	van de Putte, 2004 [61]	Low risk (high-quality randomization)	Unclear risk	Low risk	Unclear risk	Unclear risk	Low risk
	van der Heijde, 2009 [62]	Low risk (high-quality randomization)	Unclear risk	Low risk	Unclear risk	Unclear risk	Unclear risk
	Gordon, 2006 [63]	Low risk (high-quality randomization)	Unclear risk	Low risk	Unclear risk	Low risk	Unclear risk
INX							
	Kavanaugh, 2007 [64]	Low risk (high-quality randomization)	Unclear risk	Low risk	Unclear risk	Unclear risk	Unclear risk

	Koninckx, 2008 [65]	Low risk (high-quality randomization)	Low risk	Low risk	Unclear risk	Unclear risk	Unclear risk
	Mariette, 2004 [66]	Low risk (high-quality randomization)	Low risk	Unclear risk	Unclear risk	Unclear risk	Unclear risk
	Menter, 2007 [67]	Low risk (high-quality randomization)	Unclear risk	Low risk	Unclear risk	Unclear risk	Unclear risk
	Rennard, 2007 [68]	Unclear risk	Unclear risk	Unclear risk	Low risk	Low risk	Unclear risk
	Rutgeerts, 1999 [69]	Low risk (high-quality randomization)	Low risk	Low risk	Low risk	High risk	Low risk
	Reinisch, 2012 [70]	Low risk (high-quality randomization)	Unclear risk	High risk	Low risk	Low risk	Unclear risk
	Rutgeerts, 2005 [71]	Low risk (high-quality randomization)	Unclear risk	Unclear risk	Low risk	Unclear risk	Unclear risk
	Rutgeerts, 2005 [71]	Low risk (high-quality randomization)	Unclear risk	Unclear risk	Low risk	Unclear risk	Unclear risk
	Regueiro, 2016 [72]	High risk	Unclear risk	High risk	Low risk	Low risk	Unclear risk
	Sandborn, 2004 [73]	Low risk (high-quality randomization)	Unclear risk	Unclear risk	Low risk	Unclear risk	Unclear risk
	Braun, 2008 [74]	Low risk (high-quality randomization)	Unclear risk	Unclear risk	Low risk	Unclear risk	Unclear risk
ETN							
	Dijkmans, 2009 [75]	Unclear risk	Unclear risk	Low risk	Unclear risk	Unclear risk	Unclear risk
	Kloppenburg, 2018 [76]	Low risk (high-quality randomization)	Unclear risk	Low risk	Unclear risk	Unclear risk	Unclear risk
	Tyring, 2007 [77]	Low risk (high-quality randomization)	Unclear risk	Low risk	Unclear risk	Unclear risk	Low risk
	Weisman, 2007 [78]	Low risk (high-quality randomization)	Low risk	Low risk	Unclear risk	Unclear risk	Unclear risk
GLM							

	Bao, 2014 [79]	Low risk (high-quality randomization)	Low risk	Low risk	Low risk	Low risk	Unclear risk
	Deodhar, 2015 [80]	Unclear risk	Low risk	Low risk	Unclear risk	Low risk	Unclear risk
	Husni, 2020 [81]	High risk	Low risk	Low risk	Unclear risk	Low risk	Unclear risk
	Takeuchi, 2017 [82]	Low risk (high-quality randomization)	Unclear risk	Low risk	Low risk	Unclear risk	Unclear risk
	Wenzel, 2009 [83]	Low risk (high-quality randomization)	Low risk	Low risk	Low risk	Unclear risk	Unclear risk
	Emery, 2013 [84]	Low risk (high-quality randomization)	Unclear risk	Low risk	Unclear risk	Unclear risk	Low risk
	Kremer, 2010 [85]	Low risk (high-quality randomization)	Low risk	Low risk	Unclear risk	Unclear risk	Low risk
CTZ							
	Mease, 2014 [86]	High risk	Low risk	Low risk	Unclear risk	Unclear risk	Unclear risk
	Sandborn, 2007 [87]	Low risk (high-quality randomization)	Unclear risk	Low risk	Unclear risk	Unclear risk	Unclear risk
	Weinblatt, 2015 [88]	Low risk (high-quality randomization)	Unclear risk	Low risk	Unclear risk	Low risk	Low risk
	Sandborn, 2011 [89]	Low risk (high-quality randomization)	Unclear risk	Low risk	Unclear risk	Unclear risk	Unclear risk
	Loftus Jr, 2016 [90]	Low risk (high-quality randomization)	Unclear risk	Unclear risk	Unclear risk	Low risk	Unclear risk
	Yamamoto, 2014 [91]	Low risk (high-quality randomization)	Unclear risk	Unclear risk	Unclear risk	Low risk	Unclear risk

B)

		Bias due to confounding	Bias in selection of participants into study	Bias in classification of interventions	Bias due to deviation from intended interventions	Bias due to missing data	Bias in measurement of outcomes	Bi sel rep res
ADA								

	Klotsche, 2016 [92]	Probably yes	No	No information	No information	No information	Probably no	No [illegible]
	Mercer, 2017 [25]	Probably yes	Probably no	Probably no	Probably yes	No	No	No [illegible]
	Lunder, 2019 [93]	Probably no	No	Probably no	No information	No information	No	No
	Viguier, 2012 [94]	Yes	Probably no	No information	No information	No	No information	No [illegible]
	Mason, 2018 [95]	Probably yes	No	Probably yes	Probably yes	No	No information	No [illegible]
INX								
	Lunder, 2019 [93]	Probably no	No	Probably no	No information	No information	No	No
	Mok, 2014 [96]	Probably yes	No	No information	No	No	Probably no	Pr[illegible] no
	Nossent, 2001 [97]	Probably yes	No	No	No information	No information	No information	No
	Sakai, 2012 [98]	Probably no	Probably no	No	No	No information	No information	No
	Rennard, 2012 [99]	Probably yes	No	No	Probably yes	No information	No information	No [illegible]
ETN								
	Driessen, 2009 [100]	Probably yes	No information	No	Probably yes	No information	No information	No
	Lunder, 2019 [93]	Probably no	No	Probably no	No information	No information	No	No
	Mason, 2018 [95]	Probably yes	No	Probably yes	Probably yes	No	No information	No [illegible]
	Mok, 2014 [96]	Probably yes	No	No information	No	No	Probably no	Pr[illegible] no

	Sakai, 2012 [98]	Probably no	Probably no	No	No	No information	No information	No

Supplementary Figure S1: Random-Effect Funnel Plots of Interventional Studies by Drug

A) All TNF-I

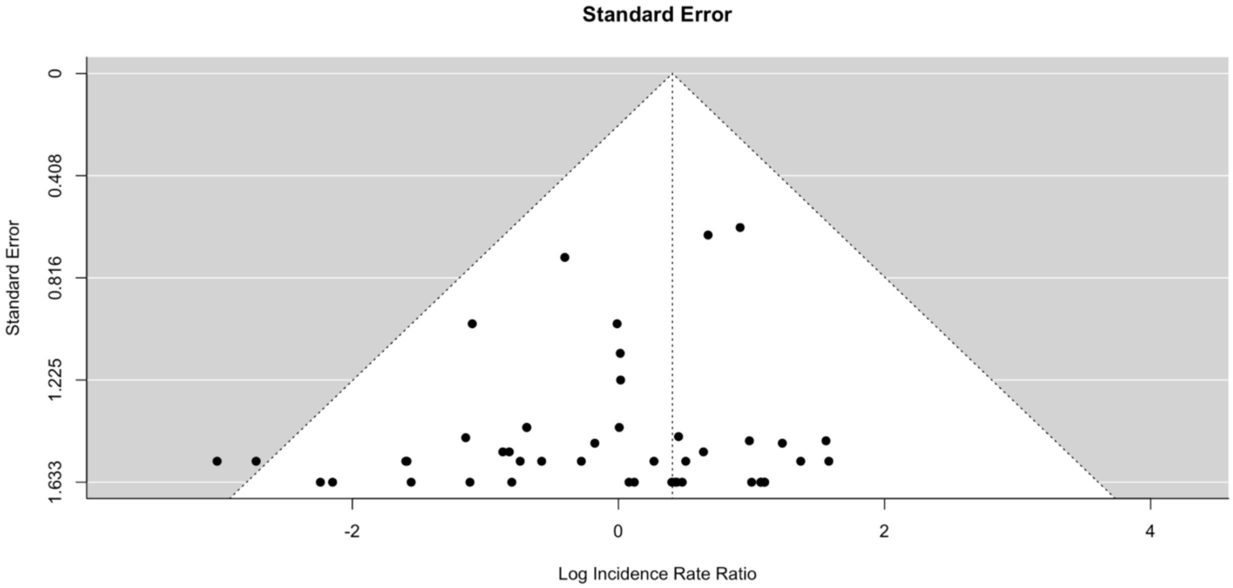

Rank Correlation Test for Funnel Plot Asymmetry: Kendall’s tau = -0.0288, p = 0.7920

B) ADA

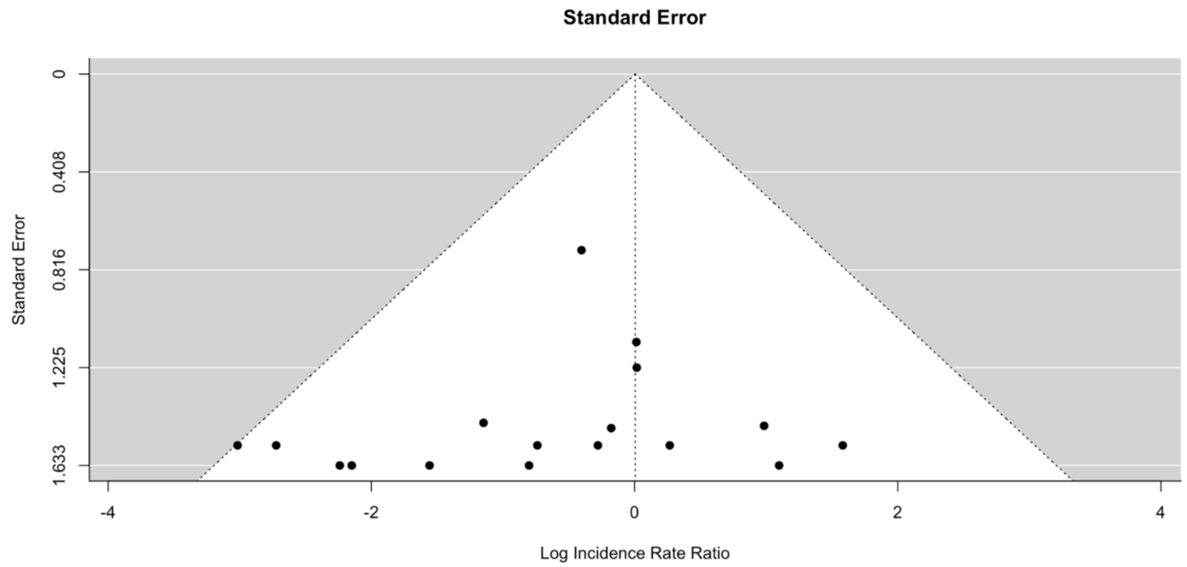

Rank Correlation Test for Funnel Plot Asymmetry: Kendall’s tau = -0.2523, p = 0.1839

C) INX

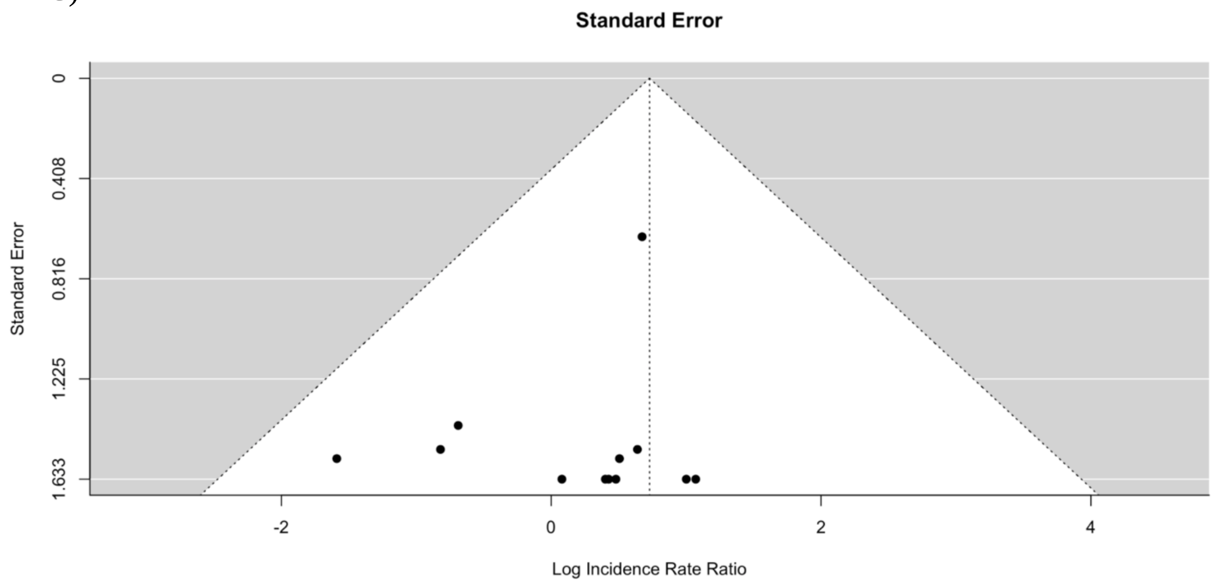

Rank Correlation Test for Funnel Plot Asymmetry: Kendall’s tau = 0.0176, p = 0.9410

D) ETN

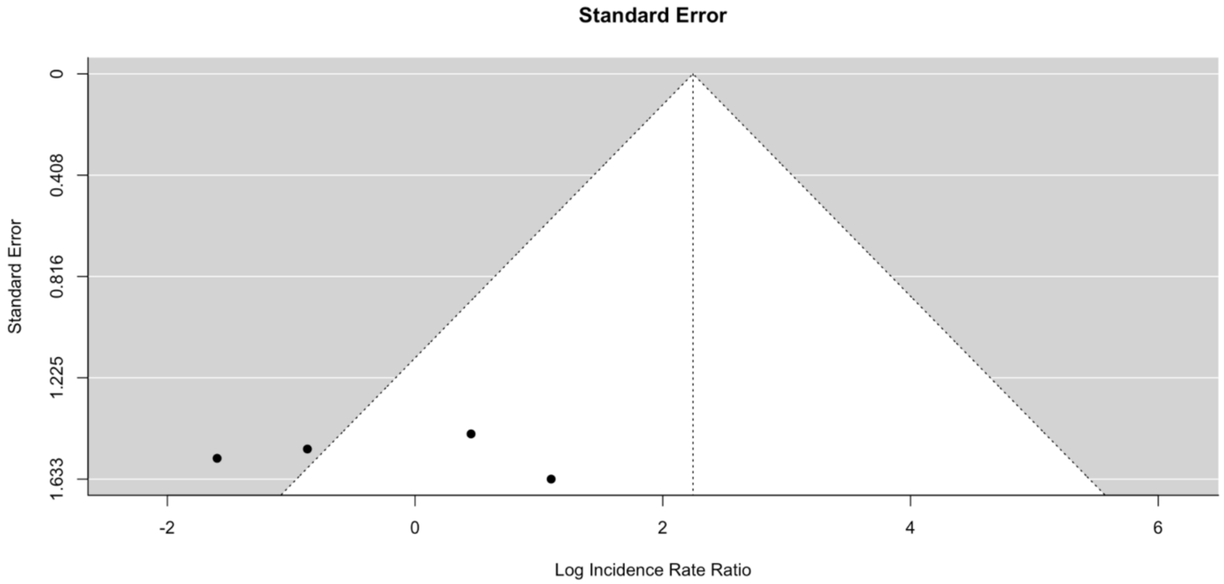

Rank Correlation Test for Funnel Plot Asymmetry: Kendall’s tau = 0.0000, p = 1.0000

E) CTZ

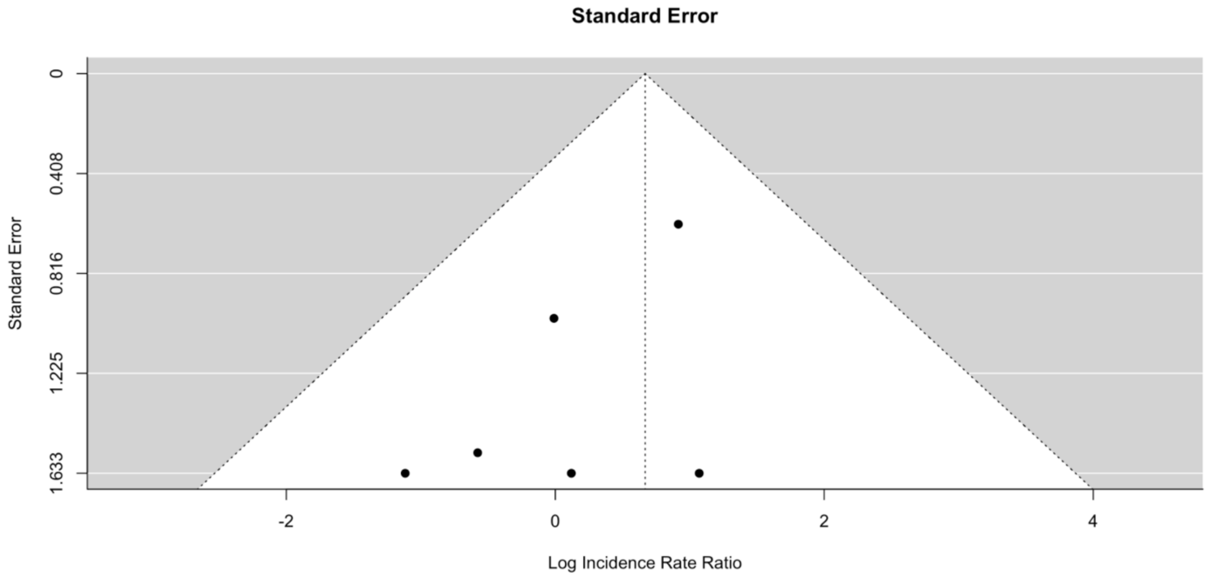

Rank Correlation Test for Funnel Plot Asymmetry: Kendall’s tau = -0.2981, p = 0.4206

F) GOL

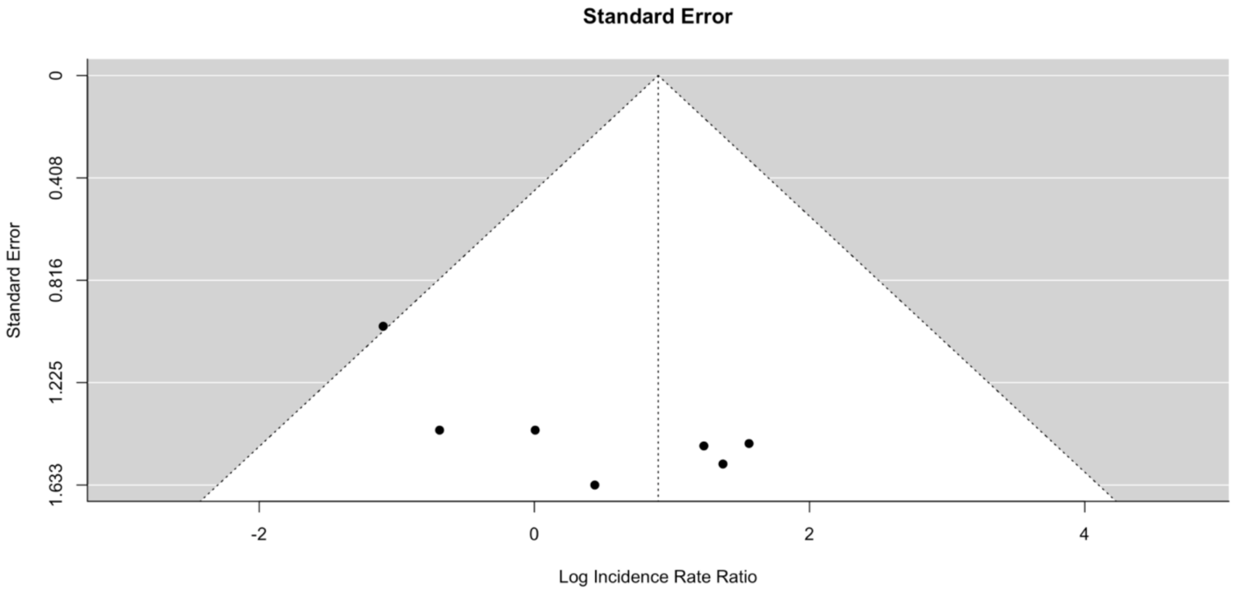

Rank Correlation Test for Funnel Plot Asymmetry: Kendall's tau = 0.4880, p = 0.1287

Supplementary Figure S2: Random-Effect Funnel Plots of Observational Studies by Drug

A) All TNF-I

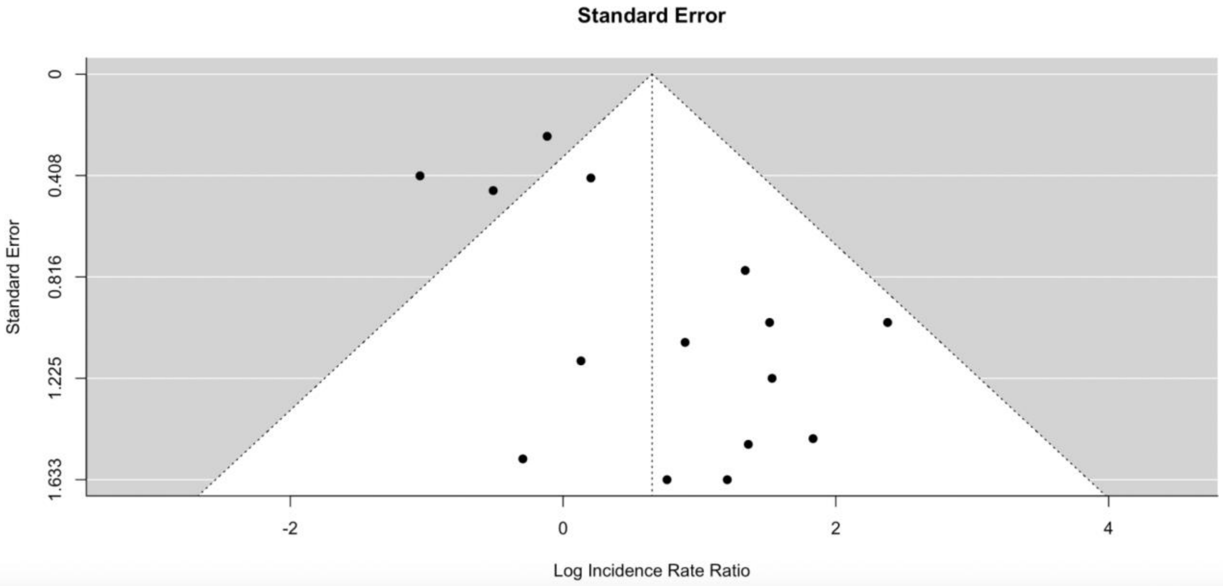

Rank Correlation Test for Funnel Plot Asymmetry: Kendall's tau = 0.0673, p = 0.7284

B) ADA

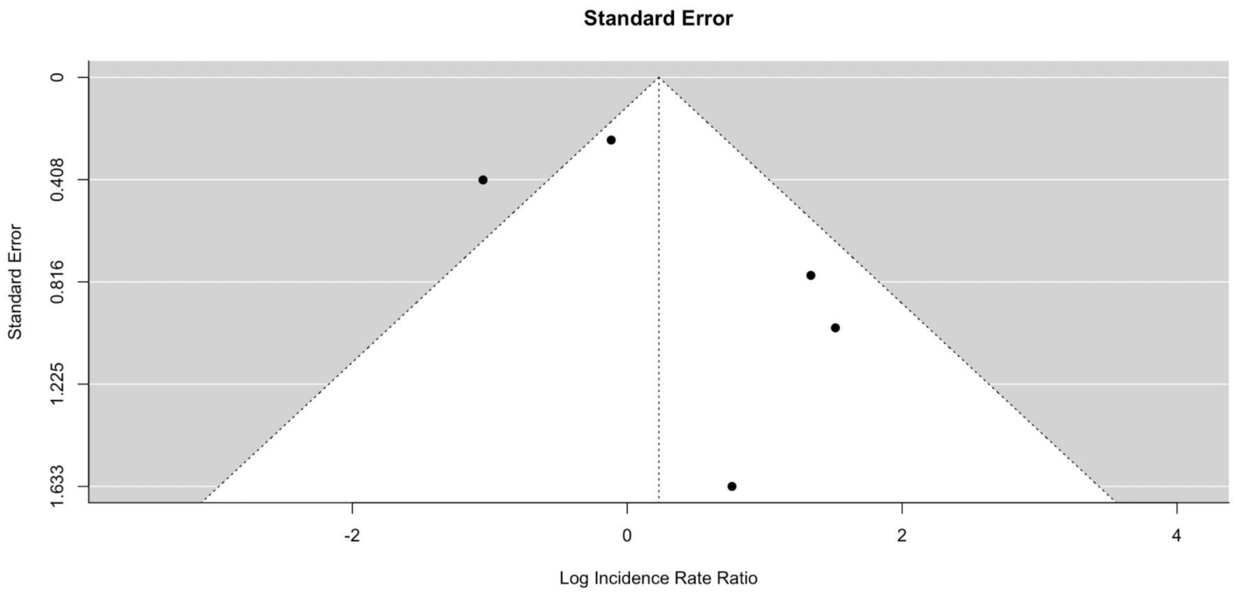

Rank Correlation Test for Funnel Plot Asymmetry: Kendall’s tau = 0.2000, p = 0.8167

C) INX

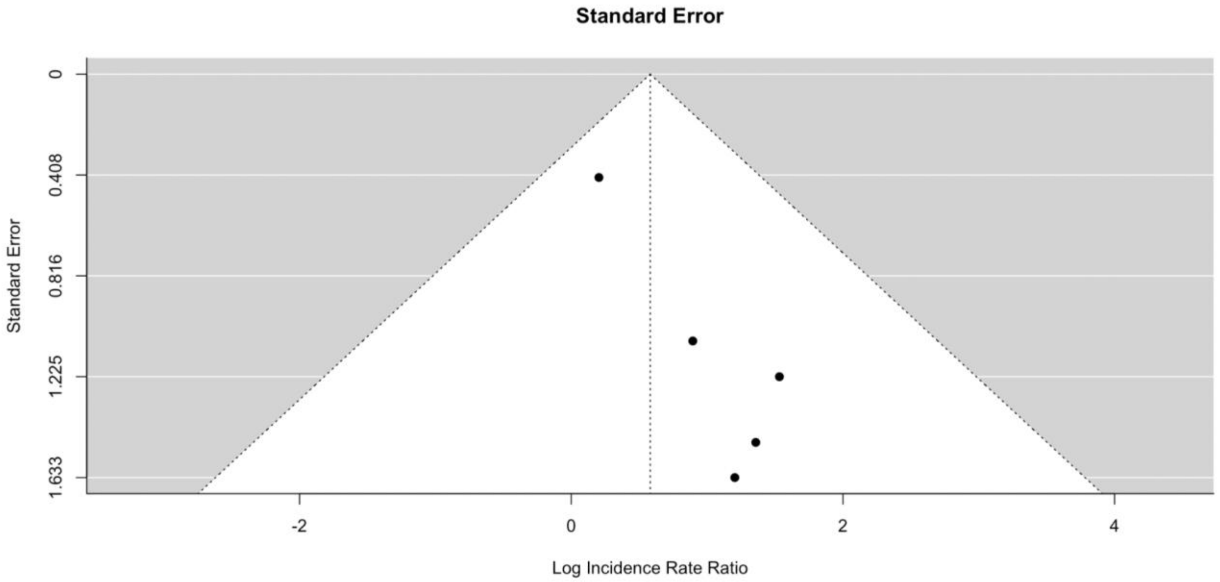

Rank Correlation Test for Funnel Plot Asymmetry: Kendall’s tau = 0.4000, p = 0.4833

D) ETN

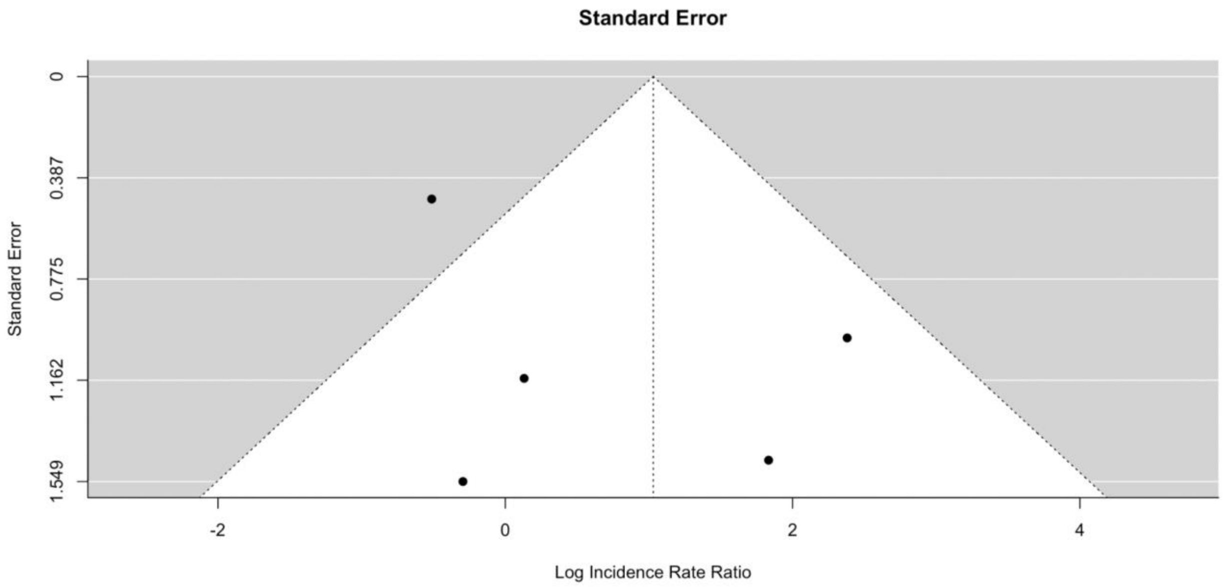

Rank Correlation Test for Funnel Plot Asymmetry: Kendall’s tau = 0.000, p = 1.000
